# Multidrug-Resistant *Salmonella* *enterica* 4,[5],12:i:- Sequence Type 34, New South Wales, Australia, 2016–2017

**DOI:** 10.3201/eid2404.171619

**Published:** 2018-04

**Authors:** Alicia Arnott, Qinning Wang, Nathan Bachmann, Rosemarie Sadsad, Chayanika Biswas, Cristina Sotomayor, Peter Howard, Rebecca Rockett, Agnieszka Wiklendt, Jon R. Iredell, Vitali Sintchenko

**Affiliations:** Westmead Hospital, Sydney, New South Wales, Australia (A. Arnott, R. Sadsad, C. Biswas, C. Sotomayor, R. Rockett, J.R. Iredell, V. Sintchenko);; The University of Sydney, Sydney (A. Arnott, R. Sadsad, C. Sotomayor, R. Rockett, J.R. Iredell, V. Sintchenko);; NSW Health Pathology, Sydney (Q. Wang, P. Howard, A. Wiklendt, V. Sintchenko);; Centenary Institute, Sydney (N. Bachmann)

**Keywords:** Salmonella, salmonellosis, antibiotic resistance, molecular epidemiology, whole-genome sequencing, genomics, public health, bacteria, antimicrobial resistance, Australia

## Abstract

Multidrug- and colistin-resistant *Salmonella enterica* serotype 4,[5],12:i:- sequence type 34 is present in Europe and Asia. Using genomic surveillance, we determined that this sequence type is also endemic to Australia. Our findings highlight the public health benefits of genome sequencing–guided surveillance for monitoring the spread of multidrug-resistant mobile genes and isolates.

Since the 1990s, the global incidence of infection with *Salmonella enterica* serotype 4,[5],12:i:- has increased sharply among humans, livestock, and poultry (*1*). This monophasic variant of *S. enterica* serovar Typhimurium ranges from pansusceptible to multidrug resistant. In 2015, an *S. enterica* strain displaying the plasmid-mediated colistin resistance *mcr-1* gene was discovered (*2*). In 2016, human and food isolates with *mcr-1* were identified in Portugal (*3*), China (*4*), and the United Kingdom (*5*). All *mcr*-1–harboring isolates were predominantly *Salmonella.* 4,[5],12:i:- multilocus sequence typing (MLST) sequence type (ST) 34. Before this study, the ST34 clone, already emerged in Europe and Asia, was yet to be detected in Australia as a drug-resistant pathogen of humans. We therefore investigated the circulation of drug-resistant *Salmonella* 4,[5],12:i:- ST34 in New South Wales (NSW), Australia.

## The Study

Since October 2016, all *Salmonella* isolates referred to the NSW Enteric Reference Laboratory (Centre for Infectious Diseases and Microbiology Laboratory Services, Pathology West, Sydney, NSW, Australia) have undergone whole-genome sequencing in addition to serotyping and multilocus variable-number tandem-repeat analysis (MLVA) performed as described (*6*). Of the 971 isolates (96% from humans, 4% from food and animals) received from October 1, 2016, through March 17, 2017, a total of 80 (8.2%) were identified as *Salmonella* 4,[5],12:i:-, and 61 (76%) of these underwent whole-genome sequencing. Five duplicate isolates were excluded. In our retrospective study, we included 54 isolates from humans and 2 isolates from pork meat obtained from independent butchers during a routine survey conducted by the NSW Food Authority in 2016.

We extracted genomic DNA by using the chemagic Prepito-D (Perkin Elmer, Seer Green, UK) and prepared libraries by using Nextera XT kits and sequenced them on a NextSeq-500 (both by Illumina, San Diego, CA, USA) with at least 30-fold coverage. We assessed genomic similarity and STs by using the Nullarbor pipeline (*7*). We identified antimicrobial resistance (AMR) genes by screening contigs through ResFinder (*8*) and CARD (https://card.mcmaster.ca) by using ABRicate version 0.5 (https://github.com/tseemann/abricate). Markers of colistin resistance were examined by using CLC Genomics Workbench (QIAGEN, Valencia, CA, USA). We identified *Salmonella* 4,[5],12:i:- genomes recovered in Europe and Asia by using Enterobase (https://enterobase.warwick.ac.uk/). We confirmed phenotypic resistance on a randomly selected subset of isolates by using the BD Phoenix system (Becton Dickinson, Franklin Lakes, NJ, USA) or Etest (bioMérieux, Marcy L’Étoile, France).

We obtained 54 isolates from 53 case-patients who had a median age of 25 years (range <1 to 90 years). We detected 20 MLVA profiles; however, 2 profiles predominated: 3-13-10-NA-0211 (45%) and 3-13-11-NA-0211 (14%). All but 2 case-patients resided in areas of distinct postal codes distributed throughout NSW; we found no apparent temporal or geographic clustering. Recent overseas travel was reported by 5 case-patients: 2 to Cambodia and 1 each to Thailand, Vietnam, and Indonesia. 

All 56 *Salmonella* 4,[5],12:i:- isolates were classified as ST34. The diversity between isolates was higher than that suggested by MLVA; we detected up to 112 single-nucleotide polymorphism (SNP) differences between isolates. The isolates from Australia clustered with each other and with isolates from the United Kingdom (Figure). Combined with the steady monthly incidence of infections, these findings suggest that local circulation of *Salmonella* 4,[5],12:i:- might play a larger role as the source of infection than independent importations from overseas. Of note, 1 isolate from pork differed from 1 isolate from a human by only 10 SNPs, indicating that pork may be a source of human infection (Figure, panel A).

**Figure:**
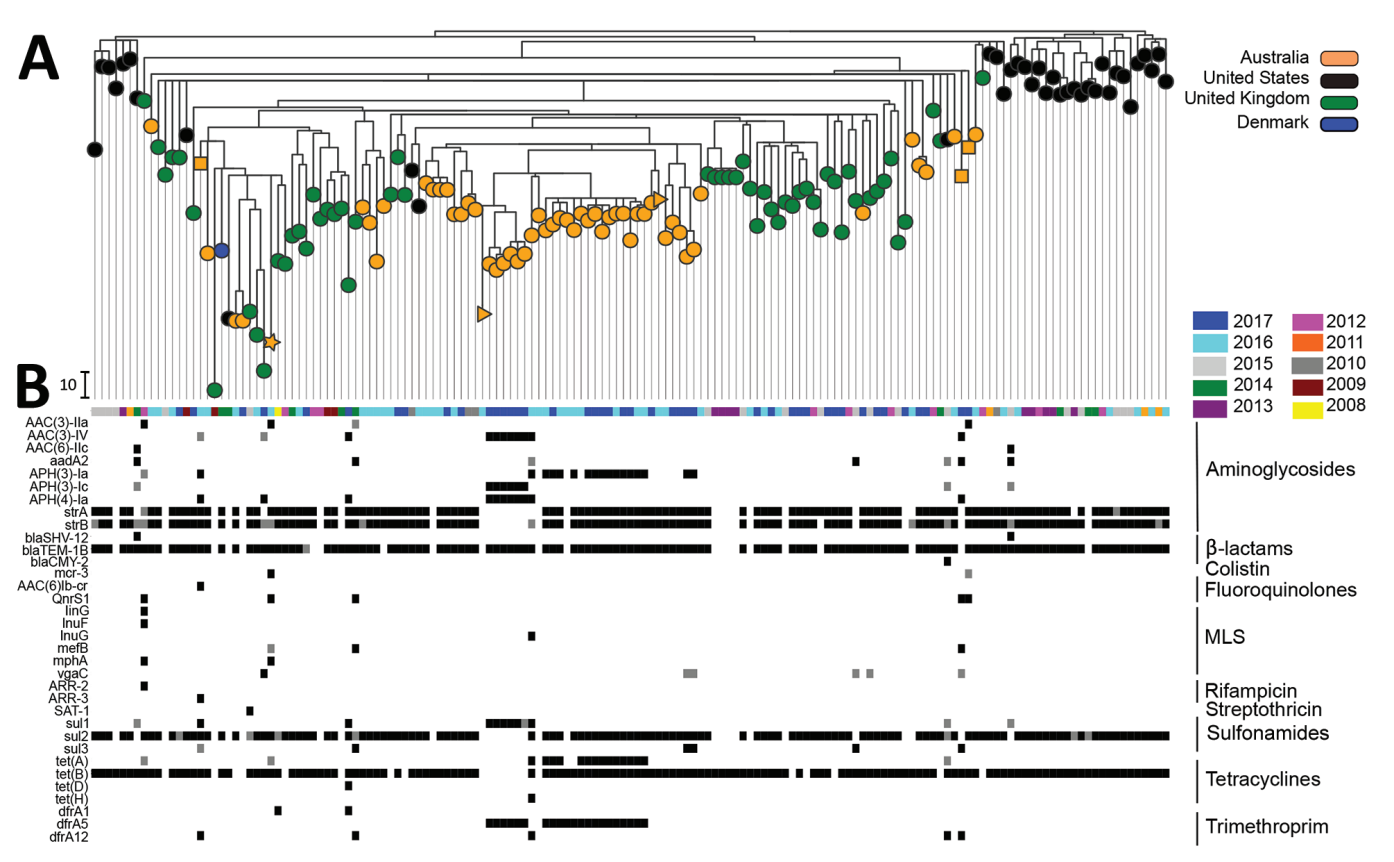
Maximum-likelihood phylogeny of whole-genome single-nucleotide polymorphisms (SNPs) of 153 *Salmonella* *enterica* 4,[5],12:i:- sequence type (ST) 34 isolates and acquired drug-resistance genes. A) SNP analysis was conducted by performing whole-genome alignment of ST34 isolates from New South Wales (NSW), Australia, and a selection of published ST34 isolates collected in the United Kingdom, United States, and Denmark by using Snippy Core (https://github.com/tseemann/snippy) (Technical Appendix). Regions of recombination were identified by using BratNextGen (www.helsinki.fi/bsg/software/BRAT-NextGen/) and removed. SNPs were identified by using SNP-sites (https://github.com/sanger-pathogens/snp-sites), and the phylogeny was generated by using FastTree (www.microbesonline.org/fasttree/). Phylogeny and antimicrobial resistance metadata were combined by using Microreact (https://microreact.org/showcase). The colistin-resistant ST34 isolate from NSW is denoted by an orange star, fluoroquinolone-resistant isolates from NSW by orange squares, and pork isolates from NSW by orange triangles. Scale bar indicates 10 SNPs. B) Year of isolation and acquisition of drug resistance. Acquired drug-resistance genes were identified by screening all isolate contigs through the ResFinder (*8*) and CARD (https://card.mcmaster.ca/*)* databases by using ABRicate version 0.5 (https://github.com/tseemann/abricate). Only genes with a 100% homology match in >1 isolate are shown. Columns depict the results for individual isolates; rows represent acquired drug-resistance genes. The antibiotic class that genes confer resistance against is indicated at right. White indicates that the specified gene was not detected, gray indicates that the specified gene was detected but sequence homology against the reference was <100%, black indicates a perfect match between the isolate and reference gene sequence. MLS, macrolide, lincosamide, and streptogramin B.

We detected AMR genes in 95% of ST34 isolates from NSW. The number of AMR genes (up to 13) was equivalent to that reported for ST34 isolates from the United States and United Kingdom (Figure, panel B). Of the 53 AMR isolates from NSW, 48 (90%) were classified as multidrug resistant on the basis of containing >4 AMR genes conferring resistance to different classes of antimicrobial drugs. Among the AMR isolates, 39 (73.5%) displayed multidrug resistance patterns, all of which are associated with resistance to aminoglycosides, β-lactams, and sulfonamides. A total of 21 (40%) isolates, including 1 from pork, had the core resistance-type (R-type) ASSuT (resistant to ampicillin, streptomycin, sulfonamides, and tetracycline) conferred by the *strA-strB*, *blaTEM*-1b, *sul2*, and *tet*(B) genes (Figure, panel B). This multidrug resistance pattern is characteristic of the European clone (*9*), which has been reported in Europe and North America and is strongly associated with pork (*10*,*11*).

R-type ASSuTTmK was found for 12 (23%) isolates from humans: genes *strA-strB*, *aph*(3*′*)-Ia, *blaTEM*-1b, *tet*(A)-*tet*(B), *sul2*, and *dfr*A5 (which confers resistance against trimethoprim). Six isolates collected from case-patients who resided in the Sydney region over a 3-week period in 2017 shared R-type ASSuTmGK: genes *aac (**3**)*-IV, *aph (**4**)*-Ia, *aph(3′)*-Ic, *blaTEM*-1B, *sul1*, and *dfr*A5 (which also confers resistance against trimethoprim) (Figure, panel B). These 6 isolates differed by 1–18 SNPs (most by <10 SNPs), and associated cases were clustered in time and occurred in neighboring suburbs, suggesting a possible cluster with a common source.

Fluoroquinolone resistance–conferring genes *qnrS1* (from 3 case-patients) and *aac(6′)lb-cr* (from 1 case-patient) were detected (Figure, panel B). As reported previously (*12*), the *aac(6′)lb-cr* (aacA4-cr) gene was plasmid borne (IncHI2 plasmid) and was typically a class 1 integron–associated gene cassette (*13*). Of these 4 case-patients, 2 reported recent travel to Indonesia and Vietnam and the other 2 had no record of recent overseas travel; hence, we could not exclude the possibility of local acquisition. The isolate from the case-patient who traveled to Vietnam also displayed resistance to colistin (MIC 4 μg/mL). Neither the *mcr-1* or *mcr-2* genes nor mutations in the *pmrAB*, *phoPQ*, and *mgrB* genes were present (*14*). Rather, resistance was conferred by a recently identified third mobile colistin resistance gene, *mcr-3*, carried on a plasmid (*15*).

## Conclusions

Using genomic surveillance, we identified the presence of novel colistin resistance gene *mcr-3* and indications that multidrug-resistant *Salmonella* 4,[5],12:i:- ST34 has established endemicity in Australia. Our findings highlight the public health benefits of genome sequencing–guided surveillance for monitoring the spread of multidrug-resistant mobile genes and isolates.

Technical Appendix Publicly available *Salmonella* sequence type 34 genomes used in study of multidrug-resistant *Salmonella* sequence type 34, New South Wales, Australia, 2016–2017.
