# Supplementary material for: Multidrug-Resistant Salmonella enterica 4,[5],12:i:- Sequence Type 34, New South Wales, Australia, 2016–2017
Source: Emerg Infect Dis. 2018 Apr;24(4):751–3. doi: 10.3201/eid2404.171619 (PMC5875280; doi:10.3201/eid2404.171619)
Supplement: Technical Appendix — Publicly available Salmonella sequence type 34 genomes used in study of multidrug-resistant Salmonella sequence type 34, New South Wales, Australia, 2016–2017. [file 17-1619-Techapp-s1.pdf]

Article DOI: https://doi.org/10.3201/eid2404.171619

Multidrug-Resistant *Salmonella* Sequence Type 34, New South Wales, Australia, 2016–2017

Technical Appendix

Technicall Appendix Table. Publically available *Salmonella* ST34 genomes used in study of multidrug-resistant Salmonella sequence type 34 in New South Wales, Australia, 2016–2017. Genomes were identified using Enterobase (https://enterobase.warwick.ac.uk/) and downloaded from the European Nucleotide Archive (ENA: http://www.ebi.ac.uk/ena). The isolate ID, year and country of collection are shown.

Isolate ID	Year of collection	Country of collection
ERR1544913	2014	Denmark
ERR24391	2009	United Kingdom
ERR24396	2009	United Kingdom
ERR24404	2009	United Kingdom
ERR24406	2008	United Kingdom
SRR1503240	2011	United States
SRR1503242	2011	United States
SRR1503243	2012	United States
SRR1503245	2013	United States
SRR1635093	2013	United Kingdom
SRR1645735	2013	United Kingdom
SRR1645745	2013	United Kingdom
SRR1646268	2013	United Kingdom
SRR1646274	2012	United Kingdom
SRR1646347	2012	United Kingdom
SRR1646348	2012	United Kingdom
SRR1646355	2012	United Kingdom
SRR1646359	2012	United Kingdom
SRR1646361	2012	United Kingdom
SRR1646365	2012	United Kingdom
SRR1646369	2012	United Kingdom
SRR1646375	2012	United Kingdom
SRR1822482	2014	United States
SRR1915431	2014	United States
SRR1915433	2014	United States
SRR1915434	2015	United States
SRR1931738	2013	United States
SRR1960281	2012	United States
SRR1966431	2014	United Kingdom
SRR1966998	2014	United Kingdom
SRR1969480	2014	United Kingdom
SRR2015055	2013	United States
SRR2015056	2013	United States
SRR2015057	2013	United States
SRR2015059	2014	United States
SRR2087755	2015	United States
SRR2152996	2015	United States
SRR2153006	2015	United States
SRR2192136	2015	United States
SRR2243429	2015	United States
SRR2243431	2015	United States
SRR2243437	2015	United States
SRR2538287	2010	Australia
SRR2538288	2010	Australia
SRR2577299	2010	United States
SRR2969505	2014	United States
SRR3098637	2015	United States
SRR3098638	2015	United States

Isolate ID	Year of collection	Country of collection
SRR3175446	2016	United States
SRR3175448	2016	United States
SRR3284675	2015	United Kingdom
SRR3284691	2015	United Kingdom
SRR3284715	2015	United Kingdom
SRR3284730	2015	United Kingdom
SRR3284731	2015	United Kingdom
SRR3284765	2015	United Kingdom
SRR3284842	2014	United Kingdom
SRR3285055	2015	United Kingdom
SRR3285406	2015	United Kingdom
SRR3322734	2015	United Kingdom
SRR3322960	2016	United Kingdom
SRR3499747	2016	United States
SRR3930234	2016	United States
SRR4032968	2016	United States
SRR4176760	2015	United States
SRR4190244	2016	United States
SRR4190251	2016	United States
SRR4450025	2011	United States
SRR4450027	2011	United States
SRR4841831	2009	United States
SRR4841833	2010	United States
SRR5193083	2016	United Kingdom
SRR5193127	2016	United Kingdom
SRR5193627	2016	United Kingdom
SRR5193644	2016	United Kingdom
SRR5193673	2016	United Kingdom
SRR5194203	2016	United Kingdom
SRR5194210	2016	United Kingdom
SRR5379273	2013	United States
SRR5631558	2017	United Kingdom
SRR5631627	2017	United Kingdom
SRR5631935	2017	United Kingdom
SRR5632032	2017	United Kingdom
SRR5632036	2017	United Kingdom
SRR5632041	2017	United Kingdom
SRR5632048	2017	United Kingdom
SRR5632062	2017	United Kingdom
SRR5632073	2017	United Kingdom
SRR5632117	2017	United Kingdom
SRR5632160	2017	United Kingdom
SRR5632173	2017	United Kingdom
SRR5632200	2017	United Kingdom
SRR5632268	2017	United Kingdom
SRR5632733	2017	United Kingdom
SRR5632860	2017	United Kingdom
SRR5632863	2017	United Kingdom
SRR5632868	2017	United Kingdom
